# Chilean Digital Press Coverage of the Relation between Diet and Mental Health

**DOI:** 10.3390/ijerph18052273

**Published:** 2021-02-25

**Authors:** Ruben Sanchez-Sabate, Esteban Zunino, Yasna Badilla-Briones, Natalia Celedon Celis, Daniel Caro Saldías

**Affiliations:** 1 Centro de Excelencia en Psicología Económica y del Consumo (CEPEC), Núcleo Científico y Tecnológico en Ciencias Sociales y Humanidades, Universidad de La Frontera, Temuco 4811230, Chile; 2 Consejo Nacional de Investigaciones Científicas y Técnicas, Instituto de Ciencias Humanas, Sociales y Ambientales, Universidad Nacional de Cuyo, Mendoza 5500, Argentina; estebanzunino@hotmail.com; 3 Departamento de Psicología, Facultad de Educación, Ciencias Sociales y Humanidades, Universidad de La Frontera, Temuco 4811230, Chile; yasna.badilla@ufrontera.cl; 4 Departamento de Salud Pública, Facultad de Medicina Universidad de La Frontera, Temuco 4811230, Chile; natalia.celedon@ufrontera.cl; 5 Dirección de Bibliotecas y Recursos de Información, Universidad de La Frontera, Temuco 4811230, Chile; daniel.caro@ufrontera.cl

**Keywords:** health communication, mass media, nutritional psychiatry, mental health, dietary patterns, food, public health, digital press, journalism, Chile

## Abstract

Chile has a serious public health problem due to the high prevalence of both unhealthy dietary patterns and mental illnesses. Given that dietary quality is positively associated with the quality of mental health, it is urgent that healthy dietary patterns be promoted among Chileans. The WHO recommends the use of mass media for the dissemination of knowledge about mental health. Since health news affect people’s attitudes and health behaviors, this study analyzed the coverage by three Chilean online newspapers with the largest readership regarding the relation between diet and mental health in 2016. A previously constructed corpus of 2551 news items about food was analyzed quantitatively. The results show that the relevance of the topic diet and mental health was low in all three newspapers. The most frequent type of information was on “foods” and not “nutrients” that “benefit”—not that “damage”—mental health. The quality of the news was poor as a narrow range of sources was found. An individual responsibility frame predominated in the information to the detriment of a public health frame.

## 1. Introduction

Chilean online press may significantly contribute to improving the Chilean population’s unhealthy diet and to reducing the country’s high prevalence of mental disorders. From a public health point of view [1], promoting habits that encourage the adoption of healthy diets and good mental health is increasingly important, especially in Chile. The latest survey on eating habits revealed that only 5% of Chileans have a healthy diet [2]. Other studies have corroborated the unhealthy diet of children [3,4], college students [5,6,7], and seniors [8]. In terms of mental health, 22% of the Chilean population have experienced some mental disorder in the last 12 months [9]. Chile is one of the OECD countries with the highest number of people with depression [10], and one of the Latin American countries with the highest levels of this pathology [11]. The number of people with dementia [12] and Parkinson’s [13] is increasing fast. And due to the COVID19 pandemic, it is predicted that the country will see an increase in the incidence, prevalence and severity of mental disorders [14], as is already happening in other Western countries [15,16,17,18]. Improving the diet of the Chilean population is a public health priority because, in addition to being the main risk factor for disease, disability and death in the world [19], it has been known already for at least a decade that there is an association between dietary quality and the high prevalence of mental disorders, even when controlling for age, socioeconomic status, education level and health behaviors like physical activity [20]. The WHO considers that the media can be an effective instrument in the dissemination of knowledge about mental health [21]. They are a factor to consider in population behaviors and in public health policies because they impact on the formation of individuals’ perceptions about health and disease [22] and they influence health attitudes (cognition and beliefs) and individual and group behaviors [23]. Therefore, this study aims to analyze the Chilean digital press’ coverage of the relation between diet and mental health.

Epidemiological studies published in the last decade have consistently shown a positive correlation between the Western diet, or diet based on highly processed foods, and psychopathological symptoms such as depression and anxiety [24]. It has been seen that certain dietary patterns have preceded the appearance of mental illnesses [24]. The inflammatory potential of a diet is associated with a higher likelihood of depression even after considering a series of sociodemographic factors, lifestyle and comorbidities [25]. In the case of children and adolescents, links have also been found between unhealthy diets and worse mental health [26]. A healthy diet, however, benefits mental health. A systematic review found a consistent trend between a good quality diet and better mental health in children and adolescents [26]. Diet can also prevent and treat mental disorders in adults [24,27]. In the case of depression, there is evidence in laboratory studies, research in population and clinical trials that endorse adherence to a healthy diet as a way to contribute to its prevention and treatment [28,29,30]. All this evidence has contributed to the development of nutritional psychiatry [31,32,33,34], a discipline that holds that nutrition is a modifiable risk factor for mental illnesses [35] and that, therefore, can affect the prevention and treatment of mental health disorders [34,36,37,38].

The spreading of this knowledge would benefit people’s health [34,39,40] and, as a result, the Chilean economy. Mental issues and disorders are the main source of disease burden [11]. Dementias are the third more expensive disease, only behind cardiovascular diseases and cancer [12]. According to the Global Burden of Disease Study (GBD) 2017, depressive disorders, headache disorders and anxiety disorders are the second, third and fifth cause of disability, respectively [41]. According to the Chilean Government, since 2008 the primary cause of medical leaves granted is mental illness, which represents more than 20% of the total cost of medical leaves [11]. In addition, it is likely that reinforcing treatments for mental illnesses with healthy diets is cost-effective in Chile, just as it is in Australia and New Zealand [42].

The mass media have a great impact on people’s ideas about nutrition [43] and mental health [44]. For example, consumers pointed to the media as the main cause for changing their attitudes and behaviors in terms of diet and nutrition [45]. This influence is partly due to the ability of the mass media to position topics—along with the way to address them—in the public sphere. From the Agenda Setting theory, it is predicted that topics disseminated by the mass media (media agenda) will become relevant topic for citizens and social actors (public agenda) [46]. From the point of view of Framing, it is argued that the media influence how society thinks about topics on the public agenda by “selecting some aspects of reality and making them more relevant in a communicative text, so that a certain definition of the problem, a causal interpretation, a moral evaluation and/or a recommendation of treatment for the described subject is promoted” [47]. In that sense, there is evidence that the mass media are a relevant actor in the construction of the public health agenda [23] because they select the health topics to be circulated—and which ones are omitted (agenda setting) [48] they shape issues from a definition of the public health situation, and they present its causes together with the agents responsible for it and its potential solutions [48,49]. It is not surprising, then, that all the stakeholders in the formulation of public health policymaking recognize the power of the media [50], since the information about health that they disseminate can support or undermine public health objectives and interventions [51] to the point that health news can have a stronger effect on the health behaviors of the population than costly institutional or governmental campaigns [22].

Due to the influence the media have on the population and on shaping political health agendas, the studies that analyze media coverage of diseases like cancer [52,53] obesity [54,55], and general health [56,57,58] abound. Studies on Spanish-speaking media have also analyzed the media coverage of cancer [59] obesity [60,61,62,63], AIDS [64], rare diseases [65], general health [66], the Mediterranean diet [67], and mental health [68,69]. To date we have not found any studies that have analyzed the coverage by the English- or Spanish-speaking media on the relation between diet and mental health. The most similar study detected is an analysis of the coverage that the Spanish press gave in the first half of 2014 to the general topic of diet and health [70].

The aim of this study is to identify and analyze the news published by three Chilean digital newspapers in 2016 that contains information on the relation between diet and mental health. In specific terms the goal is to establish: (1) the relevance of news related to diet and mental health; (2) the news sources that achieve authority in the media treatment of this topic, and (3) the type of frames included in these news items. The general and specific aims start from the following research questions that orient the development of this exploratory-descriptive study: (1) How relevant was the information on the relation between diet and mental health in the Chilean digital press? (2) What types of sources contribute to the media definition of the topic? (3) What are the main ways that the media usually frame such articles?

## 2. Materials and Methods

In relation to the proposed objectives, a methodological strategy was developed that consists of analyzing quantitative content on the news about diet and mental health published by the digital newspapers emol.com, latercera.com and elmostrador.cl in 2016.

Used since 1930 in the United States [71], the technique consists of creating a research protocol that rests on the scientific method and that starts with available data, although not in its context. This allows the analyst to make summary descriptions of messages that are highly varied in nature [72,73], to make inferences about the data in relation to their context and justify them [71].

To reach standardization in the empirical work, a series of pre-stipulated steps is needed [74]. First, a conceptual framework of the content analysis is created. Then, a universe or population is constructed based on what is meant to be studied. Third, if necessary, a sampling is performed to determine which units will be subject to coding.

For this, a previous conceptual definition of the variables and the construction of a category system are essential. This system must be exhaustive and mutually exclusive, so there are no doubts at the time of coding [72].

Fourth, a codebook is designed, an operating procedure that makes it possible to analyze the variables that arise from the research questions and hypothesis. Then, all activities performed through quality tests will be evaluated, which will allow the levels of reliability of the work to be determined. Finally, the study results and conclusions drawn are presented.

Based on the preceding methodological descriptions, a study design is established that includes different working phases with the analysis units.

### 2.1. Construction of the Corpus

The first step in the design of the methodological strategy [75] consisted of identifying the analysis units. These “are never absolute: the interaction between reality and its observer; they are a function of the empirical facts, of the purposes of the study and of the demands made by the techniques available” [71]. Consequently, these were identified from the questions to be answered.

The analysis units for the present study were identified from a previously constructed corpus of food-related news items published by emol.com, latercera.com, and elmostrador.cl in 2016 [76]. The rationale, method and results of this already constructed corpus on food in general were published in 2019 in English (translated version) [76] and Spanish (original version) [77]. A custom dictionary was built on anthropological and sociological works by a process of conceptualization of the food fact and, then, its operationalization into a list of keywords that, due to their properties, uses, and semantic meanings, are representative of the food phenomenon and operative for empirical work. Combining a CATA (computer-aided text analysis) [73] with human coding, a total of 2551 news on food published by the three aforementioned digital newspapers were identified. Emol.com (*n* = 912) and latercera.com (*n* = 957) were chosen for being the two online newspapers with the largest readership in Chile and each one respectively being the greatest exponent of the two most important communication groups in Chile. Elmostrador.cl (*n* = 682) was chosen for being the most read independent online newspaper when creating the corpus [76].

This previously constructed corpus of 2551 food-related news was used to identify those articles that reported on, or simply mentioned, some relation between diet and mental health. Coding 2551 news items was done in two stages. The first stage consisted of applying a CATA method [73]; the second was performed by 2 human coders. The first stage of the analysis was carried out with the quantitative text analysis software Wordstat 8 (Provalis Research, Québec, Canada). Two experts, a psychologist and a dietician, helped in the construction of the custom dictionary used to analyze the news items in this first stage. This decision provides reliability to the dictionary, as the instrument not only foresees the colloquial use of the discourses related to the issue, but also incorporates technical vocabulary related to the problem. From the theory and the aims of this study, the concept “diet” was defined as the “set of substances that are regularly ingested as food” [78], and the concept “mental health” was defined as “the individual’s ability to adapt to their milieu, to interact with their surroundings, to maintain a sense of purpose in life and to have a subjective sensation of well-being” [11,21,79]. The concept “diet” was divided into two categories: “food ” [80,81] and “nutrients” [82,83,84]. The concept “mental health” was divided into two categories: “mental disorders” [85,86] and “affective states” [87,88,89]. These four categories were defined and operationalized (see Table 1) in four word lists that ended up constituting an ad hoc dictionary. With this dictionary Wordstat 8 was instructed to identify the cases (news items) where there was a co-occurrence of a minimum of 1 key word from each of the two general concepts (diet and mental health). Thus, Wordstat was instructed to identify cases where one key word from either “mental disorders”/“affective states” AND one key word from either “nutrients”/“food” occurred.

Two human coders coded the news items as relevant or irrelevant. Those news items that mentioned or reported on some type of relation between diet and mental health were deemed relevant. Those news items that, although they presented co-occurrence of key words from both concepts (“diet”; “mental health”), did not mention or report on any relation between the two were deemed irrelevant. This process was carried out by two independent human coders. The cases where there was a discrepancy between coders were discussed until a consensus was reached.

### 2.2. Analysis of the Corpus

The content analysis of the news items identified as pertinent was performed by 2 human coders independently and the discrepancies were solved by consensus. The analysis of the corpus considered the following variables:

- Hierarchy of the topic: the hierarchy of the content on diet and mental health was analyzed according to the following categories:
  ○ Central topic: the relation between diet and mental health is the main topic of the news item;
  ○ Secondary topic: the information on the relation between diet and mental health appears in the news item, the central topic is the relation between diet and health in general;
  ○ Tangential topic: the information on the relation between diet and mental health appears in the news item, the central topic has nothing to do with the relation between diet and health in general;
- Authorship: The authorship of the news item was coded according to the following categories: “agency” (signed by a news agency), “corporate” (institutional signature of the newspaper company) and “signed” (carries the authorship of a journalist).
- Topics: the contents were coded on the relation between diet and mental health according to the following categories: (this analysis only was conducted with the help of QDA Miner 5 (Provalis Research, Québec, Canada), a mixed methods and qualitative data analysis software.)
  ○ Nutrients/foods benefit/detrimental to mental health: when the effects (or associations) (positive—treat, prevent, or negative—cause or increase the risk of) that foods or nutrients have on mental disorders (such as depression and anxiety, etc.) are reported.
  ○ Nutrients/foods benefit/damage the affective or emotional state: the effects (or associations) (positive or negative) that the foods or nutrients have on a person’s affective state (feelings and emotions) are reported.
  ○ Others: some type of relation between diet and mental health outside the two previous categories is reported.
- Sources: national and international official sources were coded, such as the Government and the World Health Organization (WHO); unofficial sources, and where the news item did not attribute the information on diet and mental health to any source, were coded as “unknown source”. In cases where the sources were unofficial, the following source types were considered:
  ○ Scientific sources, such as scientific papers, academics, scientific associations, among others
  ○ Nutritionists/dieticians
  ○ Health personnel like doctors and nurses
  ○ Communication media
  ○ Sources from the food sector, like the food industry
  ○ Sources from the social sector, like consumer associations, individuals
  ○ Professionals of complementary health therapies
  ○ Others
- Frames: from previous literature on the coverage of the topic of health in the press, in this study a deductive method was applied to determine the attribution of responsibility [90] operationalized into individual, collective [60], and/or public health frame [55]. In this study, the individual responsibility frame links the risk factors associated with disease or affective state with an interpellation to the reader so that they resolve the problem individually through diet. The collective responsibility frame relates the responsibility of the State, food industry or others with the need to implement political-educational intervention measures to improve the diet of Chilean society. The public health frame puts the disease or affective state in its social context, provides information on its risk factors and explains how to prevent it or treat it.

### 2.3. Questions/Variables for the Construction of Frames

#### 2.3.1. Individual Responsibility Frame

(A1) Does the news invite the reader explicitly to consider the possibility of changing eating habits according to the information on diet and mental health presented?

(A2) Are intervention measures proposed that target the individual (promotion of healthy life, education campaigns), and not that change public policies or industry practices?

#### 2.3.2. Collective Responsibility Frame

(B1) Is the role the State, food industry, schools, (public institutions), food industry plays or can play in promoting (facilitating access) or discouraging the consumption of certain foods/nutrients to benefit mental health explicitly mentioned or recognized?

(B2) Are intervention measures like public policies, industry regulations, school programs proposed…?

#### 2.3.3. Public Health Frame

(C1) Is the disease or state of mind that diet causes/increases the risk of/prevents connected to the social and environmental context of the country?

(C2) Are the risk factors associated with disease or affective state exposed? (Diet also suffices to answer yes).

(C3) Does the news item include information to prevent/remedy mental illnesses or negative affective states? (Diet also suffices to answer yes).

## 3. Results

### 3.1. News Frequency

The first stage of analysis using the CATA system yielded a total of 424 news items (emol.com (*n* = 152), latercera.com (*n* = 171) elmostrador.cl (*n* = 101)) in the co-occurrence of at least one keyword from each of the two categories included: “Diet” and “Mental Health”. The second stage of analysis completed the first specific objective of this study by identifying a total of 26 news items (25 distinct and 1 repeat signed by BBC World that appeared in two newspapers) that actually contain information on diet and mental health. This represents 1% (26/2551) of the corpus on diet analyzed. Emol.com published almost twice as many news items with information on diet and mental health than all the news items published by latercera.com and elmostrador.cl (emol.com (*n* = 17), latercera.com (*n* = 6), elmostrador.cl (*n* = 3)). However, the presence of the topic diet and mental health on the agendas of three newspapers is minimal (emol.com 1.86% (17/912), latercera.com 0.6% (6/957), elmostrador.cl 0.43% (3/682).

### 3.2. News Hierarchy

Although the frequency of news on the subject was marginal, the hierarchy of the issue of diet and mental health was high when this relation was made visible in the media (see Table 2). In 50% of the cases identified as pertinent, it was the central topic in the news, in 42% it was a secondary topic, and in 7.6% of the news the relation between diet and mental health appeared only tangentially. Emol.com granted the greatest hierarchy to the topic of diet and mental health.

### 3.3. Authorship

The three newspapers present a quite different distribution of authorship (see Table 3). All the news items except one published by emol.com (*n* = 17) have a corporate signature. Elmostrador.cl (*n* = 3) published two agency (BBC World) news items and one with a corporate signature. Latercera.com also (*n* = 6) published two news items from an agency (BBC World). Only five news items were signed by a journalist: four in latercera.com and one in emol.com. The results show that the informative content on the subject came from—or at least was seriously conditioned by—outside the newsrooms.

### 3.4. Topics

Table 3 presents the frequency in all three newspapers of each of the codes coding the effects of diet on mental health. For this coding, headline, subheadline and text of the news item were considered.

In more than half of the analyzed cases (53.8%), foods that are beneficial for the prevention and/or treatment of a mental disorder are reported. Moreover, this type of information has been coded the most (*n* = 30/27.5%), which is why it is possible to state that the topic “Foods benefit mental disorder” was the most frequent in the analyzed corpus. The relation between foods and mental disorders, either beneficial or detrimental, was the most frequent by far: this was coded a total of 49 times (45%), appearing in 14 cases (53.8%) (benefit) and 10 cases (38.5%) (detrimental), far from the second most coded type of relation, i.e., the relation between diet and affective state, coded a total of 32 times (30.2%), appearing in 10 cases (38.5%) (benefit) and 6 cases (23.1%) (detrimental). At the other end, the topic “nutrients detrimental to affective state” was only coded once (0.9%).

When comparing categories, it is observed that the frequency and presence of the category “Diet and mental disorder” is far beyond the category “Diet and affective state”. The first was coded a total of 64 times and the second a total of 44 times. This superiority holds when comparing each of the codes from the two categories. When compared by subcategories, it is observed that the frequency and presence of information on the *beneficial* effects of food/nutrients on mental health are always greater than the information on the *detrimental* effects of food/nutrients. Furthermore, the content on the effects of *foods* on mental health is more frequent and has a greater presence than the information on the effects that *nutrients* have.

### 3.5. Sources

In no news items were official sources mentioned when giving information on diet and mental health (according to the proposed operationalization, these refer to State, governmental sources or those originating from supranational organisms like the WHO). Table 4 presents the percentage of cases in which the information on diet and mental health is attributed to each one of the types of sources included:

Scientific sources appear in 50% of the news items analyzed. The second most frequent source type are health professionals (dieticians and health personnel), present in 34.6% of the cases. The overall average of sources cited by news article is 1.6. The high percentage of news items with unknown (23.1%) or media sources (26.9%) is worth noting. In the case of the media sources, it must be borne in mind that these only appear in the news published by emol.com (7 cases), the newspaper with the most cases (65.3%) in the corpus. During this study, it was observed that emol.com also publishes information reported by English-speaking newspapers, such as The Telegraph, The Huffington Post UK, and The Hippocratic Post, among others. Of the 13 cases where the central topic is the relation between diet and mental health, only in 7 is a source cited, in 2 cases 2 sources are cited, although one of them is the news media that collects from the other source, in 2 cases 2 sources are cited, in 1 case 3 sources, and in 1 case 6 different scientific studies are supposedly cited, although none is identified.

### 3.6. FRAMES

In order to establish the presence of the different frames on the topic a factorial analysis was performed on the variables stipulated in the code book in order to analyze the correlations among them and thus surmise the number and type of dominant frames in the news.

The results expressed in Table 5 on the behavior of the different frame elements reveal that in the analyzed corpus two of the three frames measured are verified with a certain consistency: (1) the individual responsibility frame, and (2) the collective responsibility frame. The public health frame does not demonstrate statistical consistency for this case, since its elements correlate more with those of the other two frames than to each other; therefore, it is ruled out as a theoretical construct for this study. However, an additional finding was produced that consists of some of the variables of the public health frame correlating significantly with the individual and collective responsibility frames, which were the two predominant ways in which the media shaped the information. The exposure to risks associated with disease or affective state (variable C2) correlates with posing the problem in individual terms, i.e., with news items that encourage the reader to change eating habits and/or that offer intervention measures aimed at the individual. Thus, registering the problem in relation to the environment and social context (variable C1) correlates with collective intervention measures that put the State at the center. In that sense, it may be concluded that there are two media frames at odds due to the definition of the problem and the attribution of responsibility that are the result of the correlation of the variables from the three frames proposed initially (individual, collective and public health). While the first of these presents the issue as an individual matter, where the responsibility for the causes or solutions falls to the subjects, the second, which is more related to a comprehensive approach to public health, registers the topic in a broader context and establishes collective and State responsibilities for its resolution.

However, on this point it is important to determine which of the two frames (individual or collective responsibility) predominated in the informative treatment. Table 6 expresses the results of the average presence of the different variables.

Among the news items analyzed, the individual responsibility frame predominates, with an average presence of 0.84. This contrasts with the low presence of the collective responsibility frame, whose average is 0.30.

## 4. Discussion

The WHO considers that the media can be an effective instrument in the dissemination of knowledge about mental health [21]. By giving information about the benefits and harms a diet can have on people’s mental health, Chilean mass media may contribute to improving society’s well-being and reducing the disease burden of mental disorders in Chile. Therefore, this study analyzed three Chilean digital newspapers’ coverage of the relation between diet and mental health. The frequency of information on the relation between diet and mental health is very low in the three digital media. The hierarchy of this theme, however, is high. The distribution of authorship is different among newspapers. The most frequent topic is “foods that are beneficial for the prevention and/or treatment of a mental disorder”. The less frequent topic is “nutrients detrimental to affective state”. The most frequent sources are scientific, first, and health professionals, second. The overall average of sources cited by news article is 1.6. Individual and collective responsibility frames have statistical consistency but not the public health frame.

The almost zero presence of the theme diet and mental health on the agendas of the Chilean online media with the largest readership contrasts with the high prevalence of unhealthy diets and mental illnesses in Chilean society [2,10], and with the remarkable social and economic damage that mental illnesses cause in Chile [9,11]. If the scientific evidence that points to diet as an effective and economical way to prevent and treat mental illnesses [24,26,27] is also taken into consideration, the low dissemination of information on the relation between diet and mental health in 2016 partly contravenes the recommendation of the WHO in the Comprehensive Mental Health Action Plan 2013–2020 to use mass media to spread information on mental health.

From the deontological point of view, journalists are called to cover social needs and affairs proportionally and comprehensively [91] for the sake of the common good [92]. The field of health communication already noted some time back the responsibility of journalists—due to their communication skills—in promoting health [93]. This duty and responsibility are supported by the facts: nowadays, the population obtains more information on health from the mass media than from health personnel [91,94]. Therefore, the coverage by the three Chilean media analyzed with respect to the relation between diet and mental health is insufficient. This scant presence of the theme on the agendas of three newspapers analyzed is comparable to the presence of diseases with low incidence. For example, the presence of acquired brain injury (ABI) in the Spanish general press with an incidence in Europe of less than 1% was scarce between 2010 and 2013 [95]. The high prevalence in Chile of unhealthy eating habits [96] and mental health problems [9] would justify a much higher presence of the relation between diet and mental health on the agenda, perhaps similar to what diseases with a high prevalence have in the West. In the case of obesity, for example, it has been seen that its presence on media agendas has increased in line with the increasing social relevance of this disease [60]. In Japan, to offer another example, the relatively high presence of breast cancer in the press is on a par with its social incidence [53].

The low frequency of news with information about the relation between diet and mental health can also be interpreted as a missed opportunity to generate a greater online readership and thus increase the economic income of the media. The interest in information on nutrition among the Western population is a fact. A study conducted by ComScore (2018) on Internet users in Germany, France, the United Kingdom, Spain and Italy found that at least 130 million people visited a diet website in 2018 [97]. It has been seen in the US that there is an increasing demand for health information [98] and that the Internet is where 59% of Americans are going to look for it [99]. We have not found such data for Chile, but considering that the average American and Spanish media have been increasing the publication of news items on health for more than a decade [100], and that in Chile the online media are the first preference for information [101], it is reasonable to anticipate that if the media analyzed published more information on diet and mental health they could attract a larger readership while at the same time fulfilling their social role with greater relevance.

Although the presence of the theme of diet and mental health was low in the Chilean press, when the investigated issue was addressed, it was the focus. In that sense, it can be stated that a low frequency was combined in this case with an important visibility of the news produced on the issue under study. Considering that frequency and hierarchy are both central factors of news relevance on media agendas, it is possible to conclude that the issue here obtained low to moderate news relevance.

The analysis of authorship allows at most to hypothesize that at least in emol.com and elmostrador.cl, there is no group of journalists specialized in health. In the case of elmostrador.cl, it could be for material reasons: it is a newspaper that only has an online version and does not belong to either of the two large Chilean media conglomerates. In the case of emol.com, the reasons for not having journalists specialized in health issues would be editorial. Emol.com is the online version of the newspaper El Mercurio, the flagship of the El Mercurio Group, one of two large communication conglomerates that comprise the Chilean duopoly [102]. If this hypothesis is confirmed, the degree of journalistic specialization on health issues at emol.com would be less than the three Spanish generalist newspapers that would be equivalent by size and readership [70]. If it is considered that the degree of specialization in health of the Spanish general press is less than desirable [70], emol.com and elmostrador.cl would have to increase the number of journalists on staff dedicated to health to publish more and better quality news items [103].

**Figure 1:**
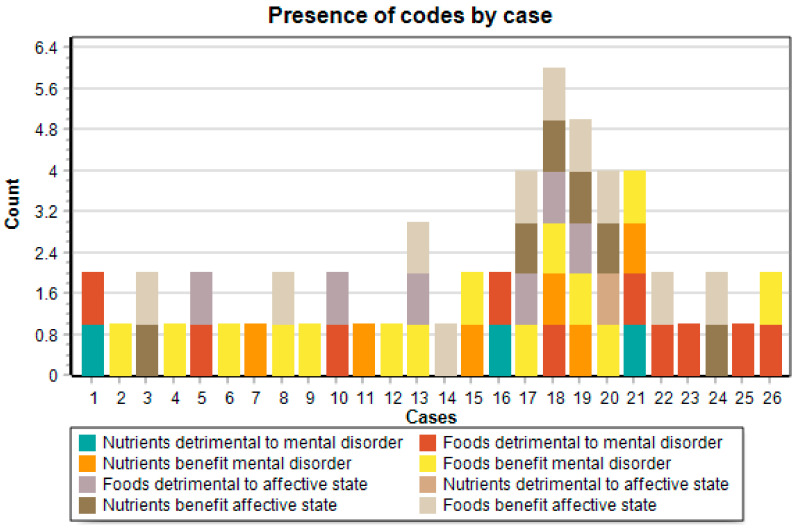
Cases where the theme of relation between diet and mental health is central: 3,4,5, 12,13,14, 17,18,19,20,21,23,24.

The analysis of the coding of the topics in light of a recent study of Spanish newspapers may indicate that an effects frame is present in the here analyzed news of the Chilean digital press. An inductive study of the news on food and health published by the three most read general newspapers in Spain identified the thematic frames used in them [70]. The effects frame—the effects that foods and diets have on health—was the most frequent in the entire corpus, and was also the most frequent in two of three Spanish newspapers studied. Considering, then, the frequency of the topic codes in the cases of our corpus where the theme diet and mental health is central (see Figure 1), it could be stated that the effects frame is also used in most of the news on diet and mental health published by the Chilean press in 2016.

The information published in these Chilean digital newspapers regarding the effects of diet on mental health fulfills recommendations that other studies on health journalism have given to increase the attractiveness of the informational contents on health. When using an effects frame, actionable information is offered, a type of information that readers demand to make decisions about their health [104]. Talking about benefits is more attractive for readers, because they see in this possible solutions to treat or prevent the negativity of a mental disorder or an undesired mood [105]. Finally, reporting on the effects of “foods” instead of “nutrients” aids in the reader’s objective and subjective understanding [106]. Objective because the reader understands better the nature of a food than a nutrient, and subjective because the reader can more easily insert the food in question in their own diet, i.e., the set of senses and dietary practices that make up their daily life.

The overall average of sources cited by news article is 1.6, far below the three sources required according to the minimum necessary number for diversity and pluralism according to the journalistic quality criteria [107]. Previous studies on the influence of sources on the credibility of health news have found that credibility depends on the type and number of sources consulted: the more expert sources, like health scientists or physicians, are quoted, the more credibility the news item has [94]. The analyzed corpus, then, has a low degree of credibility. Not all the cases quote an expert source (20/26) to support the information disseminated on diet and mental health, and in most of the news items where this is the central theme, only one source is mentioned (9/13). If the scarce presence of the theme diet and mental health on the agendas is added to this low degree of credibility, it is logical to hypothesize that the influence on the diet of Chileans of the corpus analyzed here would have been minimal.

The absence of a public health frame is a problem added to the low presence of the issue diet and mental health in the Chilean press because both social agents and individuals need to know and understand the context and the risks of any disease to promote more healthy eating habits among Chileans. A public health frame makes the reader more prepared to support changes in public policies and to adopt healthier habits [94]. Perhaps in defense of the Chilean press, it must be said that the public health frame may not be as present in the US media health news as it may be desirable. For example, such frame has been found with a low presence [108] and a medium presence [55] in the analysis of the American media treatment of obesity.

The disparity in presence found in our corpus in favor of the individual frame over the collective frame has also been encountered in studies on the coverage of obesity [54,60]. As the individual frame predominates, it is easier for the reader to perceive that the dietary changes needed to prevent risks of developing, or treating, mental illnesses can and must be solved at the level of each subject, thereby disregarding the role of the State, food industry and advertising in the formation of dietary habits among Chileans. This approach hides a complex, collective and public perspective on diet and mental health, thus conditioning “the development and implementation of public health policies or interventions” [60]. This is to say, it renders invisible the change in food policy that scientists are calling for in Western countries with the aim of improving the health of the population [109]. In addition, the predominance of an individual responsibility frame found in our corpus is consistent with previous findings. The first that is deployed from the Framing perspective highlights a trend in the media to use more episodic frames, tending to orient the information to particular, isolated and personalized cases to the detriment of more contextualized thematic approaches [110,111]. The second consists of the evidence that the media treatment of different public problems related to different types of risks also falls to a personalized and dramatic coverage that tends to present the issues much more related to the subjects’ behavior than to the social determinations of the problems [112,113], treatment that is inclined to the galvanization of moral panic scenarios [114] and to the State divesting itself of its responsibility.

### Limitations

This study has various limitations. Only the news items published by the three most important generalist newspapers online in terms of readership and media groups were analyzed. Nevertheless, Chile has a significant number of TV stations, radio stations, national and regional newspapers, as well as online news media outlets. Therefore, the results of this study cannot be generalized to the rest of the Chilean media ecosystem. Another limitation is that only news items published in 2016 were analyzed. This prevents any type of trend in the presence of the topic diet and mental health from being detected on the agendas of the Chilean online press. Moreover, having studied the coverage of such a specific subject as diet and mental health is a limitation: some of these study results, such as the analysis of the frames and the sources for example, could serve at most to generate hypotheses about the coverage of nutrition and health by the Chilean press. The low number of cases that fulfilled the inclusion criteria is also a limitation, since it prevents greater statistical generalizations. Finally, due to the impact that the COVID-19 pandemic is already having on people’s mental health, it is necessary for new analyses of the media coverage on diet and mental health at least during 2020 and 2021 to be performed. Moreover, it would be necessary to broaden the sample of Chilean media studied to include not only TV, radio and printed general newspapers, but also the media specialized in disseminating information on nutrition and/or health. Diagnoses of reality, however, are just a first step. Studies on how to increase the influence of nutrition and mental health news on people’s diet are a second step very much needed to help health journalists to better inform and educate their audiences on the relation between diet and mental health.

## 5. Conclusions

The Chilean general digital press did not contribute in 2016 to shining a light on the positive association between dietary quality and the quality of mental health. The relevance given to the topic was low, the quality of the information disseminated was low also due to the absence of numerous and diverse sources (although the scientists stood out) and specialized journalists. Nor did the Chilean digital press contribute to placing the relation between diet and mental health on the country’s political agenda.

As argued, the media are central actors in the visibility of problems that people consider important. Poor diet constitutes a factor of objective problems for individual health that, as argued in this paper, have a full impact on major health problems that imply specific demands for the health system and large budgets spent for the resolution of diseases. However, there is no direct relationship between the objective weight of this situation for public health and its media treatment. The problem here analyzed is underrepresented in the news and, it is possible to think, that this translates to a distorted perception of the importance of the issue for people.

Additionally, the lack of journalists specialized in health capable of producing technically specialized content in the Chilean media, the low quantity and diversity of sources consulted and the shallowness of journalistic reports express a low standard of information quality that contributes to compound the problem. This is complemented by the prevalence of an episodic frame [111] that hold individuals responsible both for their health deficits and for solving their problems individually as well. By framing most of the information as a subject of individual responsibility, the online newspapers have reinforced the notion that the necessary dietary changes in the majority of the Chilean population depend basically on the will of each individual, thus overshadowing the responsibility of the State, food industry and the media themselves in the actual configuration of Chileans’ dietary patterns.

Given the serious public health problem that Chile has in terms of diet and mental health, the Chilean digital press faces the challenge of making the positive association between diet and mental health relevant proportional to the prevalence of poor-quality diets and mental illnesses among Chileans, and proportional to the high disease burden that mental illnesses pose for the Chilean economy.

The findings of this work contribute to expose a picture of the relationship between public health and the media. However, the problem is broader. As stated by the WHO, States should consider the design of public policies that include a communicational dimension, according to the central role of the media in dissemination and divulgation. In this way, the media would not be the only ones responsible for the individual framing of the problem. The lack of active public health policies that approach the problem from a social, holistic, and multidimensional perspective is consistent with journalistic discourses on the problem. Meanwhile, the responsibility of the food industry should not be set aside. As mentioned, individual responsibility for health must be reversed by all actors, media included, under the guidance of the State as the central responsible for the design of public policies.

## Figures and Tables

**Table 1:** Conceptualization and operationalization of the variables.

Concept	Categories	Definition	Operationalization
Diet	Food	Set of substances that living beings eat or drink to subsist	Set of foods of plant and animal origin native to the West, as well as Chilean sweets and fast food.
	Nutrients	Chemical elements that foods contain and that are necessary for the vital functions of human cells.	Set of macro and micronutrients.
Mental health	Mental disorders	Syndromes or psychological patterns subject to clinical interpretation associated with distress or disability.	“Set of mental illnesses described in the DSM-5 [85] complemented by the CIE10 [86].”
	Affective states	Moods, feelings and emotions [89].	“Feelings and emotions related to: “Energy”, “Fatigue”, “Stress”, “Anxiety”, “Depression”, “Relaxation-well-being” [87,88].

DSM-5: Diagnostic and Statistical Manual of Mental Disorders; CIE10: Clasificación estadística internacional de enfermedades y problemas relacionados con la salud; World Health Organization.

**Table 2:** Thematic hierarchy by medium.

	Emol.com		Latercera.com		Elmostrador.cl	
Main topic	9	52.9%	3	50%	1	33.3%
Secondary topic	7	41.1%	3	50%	1	33.3%
Tangential topic	1	5%	0	0%	1	33.3%
Total	17	100%	6	100%	3	100%

**Table 3:** Frequency of codes.

Diet and Mental Health						
Category	Subcategory	Code (Topic)	Times Coded	% of Times It Was Coded	Nº of Cases in Which It Was Coded	% of Cases in Which It Was Coded
Diet and mental disorder	Diet detrimental to mental disorder	Nutrients detrimental to mental disorder	4	3.7	3	11.5
		Foods detrimental to mental disorder	19	17.4	10	38.5
	Diet benefits mental disorder	Nutrients benefit mental disorder	11	10.1	6	23.1
		Foods benefit mental disorder	30	27.5	14	53.8
Diet and affective state	Diet detrimental to affective state	Nutrients detrimental to affective state	1	0.9	1	3.8
		Foods detrimental to affective state	8	7.3	6	23.1
	Diet benefits affective state	Nutrients benefit affective state	11	10.1	6	23.1
		Foods benefit affective state	25	22.9	10	38.5

**Table 4:** Frequency of sources.

Code	Nº of Cases in Which It Was Coded	% of Cases in Which It Was Coded
Scientific sources	13	50.0%
Nutritionists, dieticians	4	15.4%
Health professionals	5	19.2%
Communication media	7	26.9%
Food sector sources	1	3.8%
Social sector sources	1	3.8%
Professionals of complementary health therapies	0	0%
Others	0	0%
Unknown source	6	23.1%

**Table 5:** Factorial analysis and correlation among the different frame elements.

Rotated Component Matrix			
	Component		
	1	2	3
Does the news explicitly invite the reader to consider the possibility of changing eating habits according to the information on diet and mental health presented?	0.941	−0.173	0.034
Are intervention measures proposed that target the individual (promotion of healthy life, education campaigns), and not that change public policies or industry practices?	0.783	0.212	−0.137
Is the role the State, food industry, schools, (public institutions), food industry plays or can play in promoting (facilitating access) or discouraging the consumption of certain foods/nutrients to benefit mental health mentioned explicitly or recognized?	0.161	0.828	0.133
Are intervention measures like public policies, industry regulations, school programs proposed?	−0.244	0.941	−0.037
Is the disease or mood that diet causes/increases the risk of/prevents connected to the social and environmental context of the country?	0.394	0.449	−0.549
Are the risk factors associated with disease or affective state exposed?	0.887	−0.037	−0.142
Does the news item include information to prevent/remedy mental illnesses or negative affective states?	−0.033	0.172	0.890
Extraction method: Principal components analysis. Method of rotation: Varimax with the Kaiser normalization.			
a. The rotation has converged in 4 iterations.			

**Table 6:** Average presence of frame elements.

	Does the news explicitly invite the reader to consider the possibility of changing eating habits according to the information on diet and mental health presented?	Are intervention measures proposed that target the individual (promotion of healthy life, education campaigns), and not that change public policies or industry practices?	Is the role the State, food industry, schools, (public institutions), food industry plays or can play in promoting (facilitating access) or discouraging the consumption of certain foods/nutrients to benefit mental health mentioned explicitly or recognized?	Are intervention measures like public policies, industry regulations, school programs proposed…?	Is the disease or affective state that diet causes/increases the risk of/prevents connected to the social and environmental context of the country?	Are the risk factors associated with disease or affective state exposed?	Does the news item include information to prevent/remedy mental illnesses or negative affective states?
Medium	0.85	0.92	0.08	0.15	0.69	0.77	0.92
*N*	13	13	13	13	13	13	13
Standard deviation	0.376	0.277	0.277	0.376	0.480	0.439	0.277

## Data Availability

No new data were created or analyzed in this study. Data sharing is not applicable to this article.
